# Histopathological Complexities and Diagnostic Challenges in Familial Adenomatous Polyposis: A Case Report

**DOI:** 10.7759/cureus.73052

**Published:** 2024-11-05

**Authors:** Ali Z Ansari, Srihita Patibandla, Sahar Hafeez

**Affiliations:** 1 Department of Pathology and Laboratory Medicine, William Carey University College of Osteopathic Medicine, Hattiesburg, USA; 2 Department of Internal Medicine, Trinity Health Grand Rapids, Grand Rapids, USA

**Keywords:** colonic adenomas, colonoscopy and polypectomy, colorectal polyp, familial adenomatous polyposis, familial transmission, fundic gland polyp, hyperplastic polyp, low-grade dysplasia, oxyntic gland adenoma, young adult male

## Abstract

Familial adenomatous polyposis (FAP) is a rare inherited disorder characterized by numerous adenomatous polyps throughout the colon and rectum, leading to a significantly increased risk of colorectal cancer. We present the case of a 19-year-old male patient with a known family history of FAP who presented with recurrent lower abdominal pain, altered bowel habits, and rectal bleeding. Initial examination identified rectal polyps, and subsequent colonoscopy revealed multiple adenomatous polyps. Histopathological analysis initially raised the possibility of oxyntic adenomas due to the glandular tissue's anastomosing and cribriform pattern. However, further detailed evaluation, which identified a significant population of both chief and parietal cells within the glandular pits, supported the diagnosis of mixed hyperplastic/fundic gland polyps with focal low-grade dysplasia. This case highlights the diagnostic complexities in distinguishing between oxyntic adenomas and mixed hyperplastic/fundic gland polyps in FAP patients, emphasizing the importance of accurate diagnosis in guiding management, including surveillance, surgical intervention, genetic counseling, and lifestyle modifications.

## Introduction

Familial adenomatous polyposis (FAP) is a rare but significant autosomal dominant genetic disorder that predisposes individuals to the development of numerous adenomatous polyps throughout the colon and rectum [1]. This condition is primarily caused by germline mutations in the adenomatous polyposis coli (APC) gene, a critical regulator of the Wnt signaling pathway, which plays a pivotal role in cellular proliferation and apoptosis [2]. The dysregulation of this pathway leads to the unchecked growth of colonic epithelial cells, forming polyps that ultimately increase the risk of colorectal cancer to nearly 100% if left untreated [1]. The onset of FAP is typically early, with affected individuals often presenting with symptoms in their teenage years or early adulthood [3]. The pathological hallmark of FAP is the presence of numerous adenomatous polyps in the colon and rectum [1]. These polyps vary in morphology, including tubular, tubulovillous, and villous types, each associated with different degrees of dysplasia and cancer risk [4]. Tubular adenomas are the most common and generally carry a lower risk of malignancy compared to tubulovillous and villous adenomas, which have a higher propensity for dysplastic changes and cancer progression. Histopathological examination reveals a progression from mild dysplasia to severe dysplasia and eventually carcinoma in situ, highlighting the critical need for early and vigilant surveillance in FAP patients [1].

In addition to colonic manifestations, FAP can be associated with several extracolonic findings, including gastric fundic gland polyps and duodenal adenomas [1]. Gastric fundic gland polyps are often found in FAP patients and are typically classified into hyperplastic, fundic gland, and oxyntic types [5]. Distinguishing between these types can be challenging, particularly when differentiating between oxyntic adenomas and mixed hyperplastic/fundic gland polyps. Oxyntic adenomas are characterized by the presence of anastomosing and cribriform glandular patterns and are typically associated with chronic hypergastrinemia [1]. Conversely, mixed hyperplastic/fundic gland polyps present with a mixture of chief and parietal cells and may exhibit focal dysplasia [1]. Accurate histological evaluation is crucial to differentiate between these entities, as the management strategies and associated risks vary significantly. The diagnosis of FAP requires a comprehensive approach that includes clinical evaluation, genetic testing, and histopathological assessment. Genetic testing for APC gene mutations provides definitive confirmation of the diagnosis and helps identify at-risk family members [6]. Endoscopic surveillance and biopsy of polyps are essential for monitoring disease progression and detecting early dysplastic changes [6].

## Case presentation

A 19-year-old Caucasian male patient with a body mass index of 24.61 presented to the emergency department with a chief complaint of recurrent lower abdominal pain, accompanied by changes in bowel habits and occasional episodes of rectal bleeding over the past several months. The patient reported that the abdominal pain, which he initially noticed approximately a year ago, had been intermittent and mild but had progressively intensified in frequency and severity over the past three months. He described the pain as a dull, aching sensation, predominantly localized to the lower quadrants, with occasional sharp exacerbations. The patient also noted a recent shift in his bowel habits, characterized by periods of constipation alternating with loose stools. The rectal bleeding, which he described as bright red and occurring intermittently, was initially dismissed by the patient as a result of hemorrhoids or dietary changes. The patient attributed his symptoms to the increased stress of school and alterations in his diet, as his busy academic schedule had led to a higher intake of processed foods and a noticeable decrease in the consumption of fruits and vegetables. He disclosed a family history of FAP, which raised concern for a possible hereditary etiology underlying his symptoms.

Upon examination, the patient appeared pale and exhibited signs of fatigue, though he remained hemodynamically stable. His vital signs were within normal limits, with a heart rate of 78 beats per minute, blood pressure of 120/80 mmHg, a respiratory rate of 16 breaths per minute, and an oral temperature of 98.6°F (37°C). The abdominal examination revealed tenderness upon palpation, particularly in the lower quadrants, with the left lower quadrant being the most affected. There was no evidence of guarding, rigidity, or rebound tenderness, and no palpable masses or indications of hepatosplenomegaly were noted. Bowel sounds were present and normoactive across all quadrants.

During the rectal examination, the external anal sphincter tone was found to be normal, with no visible evidence of hemorrhoids or fissures upon external inspection. The initial digital rectal examination revealed an empty rectal vault with no palpable masses. However, upon further and more thorough exploration, multiple firm, irregular masses were detected within the rectum, raising concern for the presence of polyps. Although nontender to palpation, these masses exhibited a notably irregular texture, which further heightened suspicion. A subsequent proctoscopy was performed, revealing a slightly inflamed rectal mucosa with areas of erythema surrounding the polyps, consistent with local irritation or inflammation. Given the clinical findings, a guaiac test was conducted, yielding a positive result for occult blood in the stool.

Due to the concerning rectal examination findings suggestive of polyps, along with the patient's notable family history of FAP, the patient was admitted for comprehensive evaluation and management. Initial laboratory investigations were conducted, including a complete blood count (CBC) and a comprehensive metabolic panel (CMP), as shown in Table 1. The CBC revealed decreased hemoglobin, hematocrit, and mean corpuscular volume levels, consistent with mild microcytic anemia. The CMP results were unremarkable, showing normal electrolyte levels, renal function, and liver enzyme activity. Given the high suspicion of FAP based on the patient's clinical presentation and family history, genetic testing for APC gene mutations was ordered.

**Table 1:** Summary of laboratory results. Pertinent findings include decreased hemoglobin, hematocrit, and mean corpuscular volume, all indicative of mild microcytic anemia

Test	Observed value	Reference range	Status
White blood cells	9.1 × 10³/μL	4.0-11.0 × 10³/μL	Normal
Red blood cells	4.1 × 10⁶/μL	4.0-5.0 × 10⁶/μL	Normal
Hemoglobin	11.5 g/dL	12.1-15.1 g/dL	Decreased
Hematocrit	35%	42%-52%	Decreased
Mean corpuscular hemoglobin	30 pg/cell	27-31 pg/cell	Normal
Mean corpuscular hemoglobin concentration	33 g/dL	33-36 g/dL	Normal
Mean corpuscular volume	78 fL	80-100 fL	Decreased
Platelet count	296 × 10⁹/L	150-450 × 10⁹/L	Normal
Mean platelet volume	9 fL	8-12 fL	Normal
Red cell distribution width	15%	12%-15%	Normal (upper limit)
Sodium	138 mmol/L	135-147 mmol/L	Normal
Potassium	3.7 mmol/L	3.5-5.0 mmol/L	Normal
Chloride	102 mmol/L	96-106 mmol/L	Normal
Carbon dioxide	25 mmol/L	23-29 mmol/L	Normal
Blood urea nitrogen	3.1 mmol/L	2.1-8.5 mmol/L	Normal
Creatinine	0.9 mg/dL	0.7-1.3 mg/dL	Normal
Glucose	84 mg/dL	70-100 mg/dL	Normal
Calcium	9.6 mg/dL	8.5-10.2 mg/dL	Normal
Albumin	4.3 g/dL	3.5-5.5 g/dL	Normal
Alkaline phosphatase	97 U/L	44-147 U/L	Normal
Alanine aminotransferase	33 U/L	7-56 U/L	Normal
Aspartate aminotransferase	28 U/L	5-40 U/L	Normal
Total bilirubin	0.5 mg/dL	0.3-1.0 mg/dL	Normal
Total protein	7.2 g/dL	6.0-8.3 g/dL	Normal
Globulin	2.3 g/dL	2.0-3.5 g/dL	Normal
Lipase	81 U/L	10-140 U/L	Normal

To address the gastrointestinal bleeding and manage the patient's symptoms, intravenous fluids were administered, and packed red blood cells were transfused to correct the anemia. Additionally, a proton pump inhibitor was started to decrease gastric acid secretion and facilitate healing any potential erosions or ulcers that might have contributed to the bleeding. An urgent consultation with gastroenterology was requested, and an emergent colonoscopy was performed to investigate the rectal polyps further. During the colonoscopy, numerous adenomatous polyps were identified throughout the colon. Multiple polyps ranging from 0.5 to 2 cm in diameter were observed in the sigmoid colon, proximal to the rectum. All adenomatous polyps identified were in the colon and exhibited a range of sizes and morphologies, including sessile, pedunculated, and cauliflower-like forms, consistent with typical colonic adenomas. As the colonoscope was advanced proximally, additional polyps were encountered in the descending colon, transverse colon, and ascending colon. The total number of polyps exceeded 20, displaying a range of sizes and morphologies. Some polyps had a cauliflower-like appearance, indicative of advanced dysplasia, while others were smaller and more indolent. Biopsy samples were collected from several representative polyps for comprehensive histopathological evaluation.

**Figure 1:**
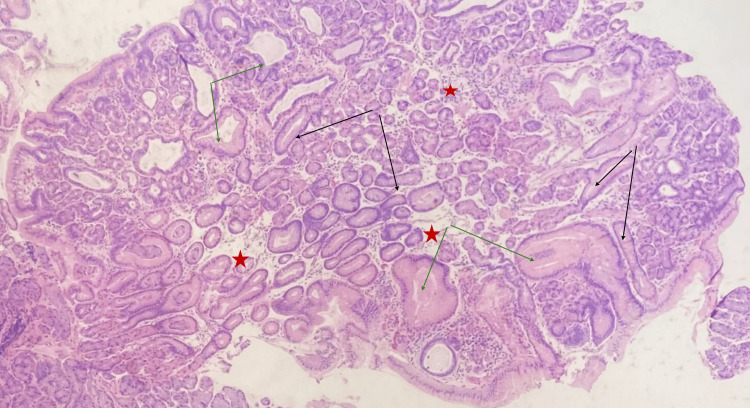
Photomicrograph at 40× magnification of the excised specimen showing multiple stellate-shaped (black arrows) and cystically dilated (green arrows) glands with edematous stroma (red stars)

**Figure 2:**
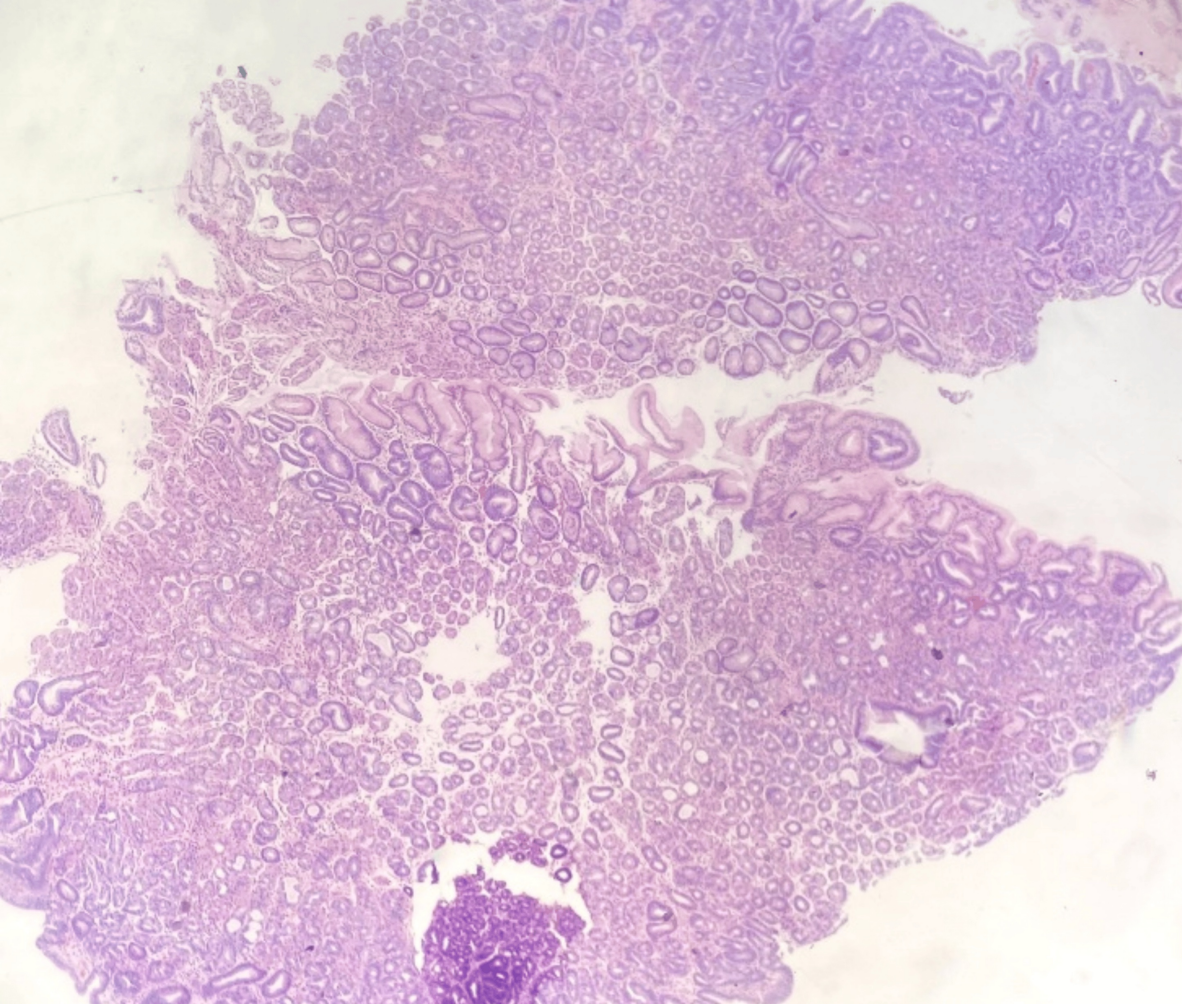
Alcian blue-/PAS-stained specimen showing a "floater" stained with Alcian blue PAS: periodic acid-Schiff

**Figure 3:**
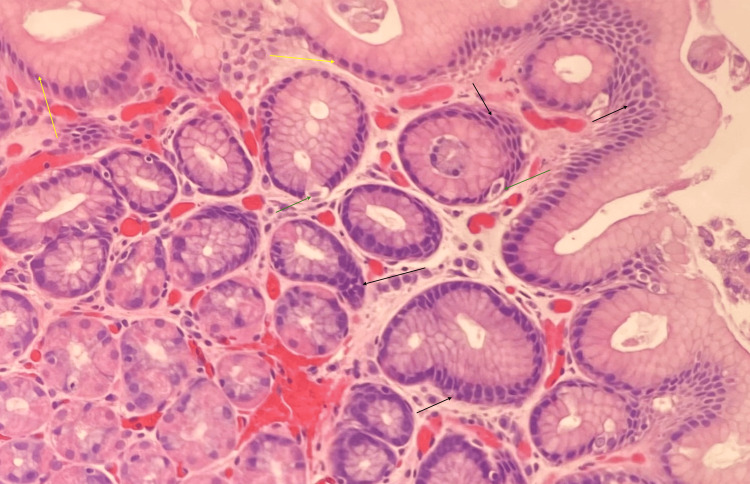
Photomicrograph at 400× magnification of polyp glandular epithelium showing modestly increased nuclear stratification (black arrows), occasional epithelial cell mitosis (green arrows), and mild nuclear stratification near the surface (yellow arrows)

The biopsy specimen obtained during the colonoscopy consisted of a segment of gastric mucosa with diverse glandular structures. Microscopic examination revealed the presence of glands with stellate-shaped luminal contours and cystically dilated glands containing both chief and parietal cells (Figure 1). An Alcian blue/periodic acid-Schiff (PAS) stain demonstrated a rare positive staining gland, identified as an extraneous "floater." At the same time, the majority of the tissue exhibited negative Alcian blue staining (Figure 2). As a precaution, *Helicobacter pylori* testing was performed, yielding negative results. This incidental gastric sample did not influence the final diagnosis, as all adenomas were confirmed to be colonic in origin. Further analysis of deeper tissue layers revealed modestly increased nuclear stratification, with occasional mitotic figures in the epithelial cells and mild nuclear stratification near the surface (Figure 3). The PAS stain also highlighted the variable prominence of the retained basement membrane, reflecting the diverse nature of the glandular changes observed.

**Figure 4:**
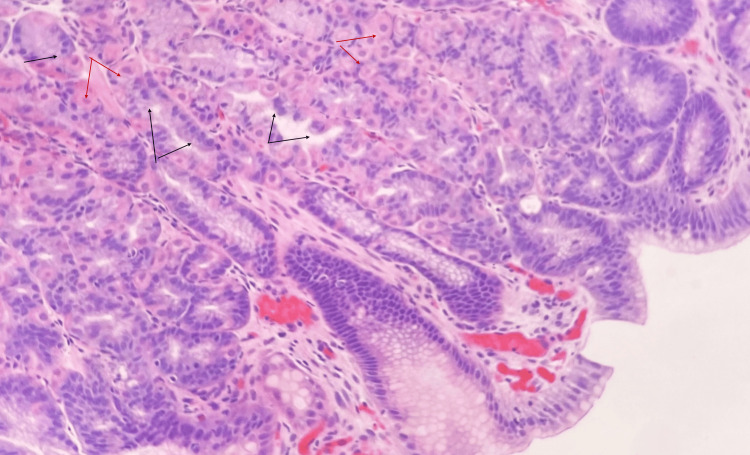
Photomicrograph at 200× magnification showing a mixed fundic gland-like appearance, featuring an equal population of chief cells (black arrows) with basophilic cytoplasm and oxyntic cells (red arrows) with eosinophilic cytoplasm

Initially, oxyntic adenomas were considered due to the anastomosing and cribriform patterns observed in the glandular tissue (Figure 1). However, a comprehensive histopathological analysis of the biopsy revealed glandular pits containing a notable population of both chief and parietal cells (Figure 4). This detailed examination, which involved evaluating the morphological features and considering the patient's clinical history, ultimately led to a diagnosis of mixed hyperplastic fundic gland polyps with focal low-grade dysplasia. The diagnosis was supported by the distinct histological patterns and the patient's background of FAP, which aligns with the development of hyperplastic fundic gland polyps rather than oxyntic adenomas.

Following the initial diagnostic workup and the initiation of treatment, the patient's symptoms showed significant improvement. He was subsequently discharged from the hospital with detailed instructions for ongoing care and close follow-up with the gastroenterology clinic. To ensure comprehensive management and risk assessment, genetic counseling was arranged for the patient and his at-risk family members. This counseling included scheduling genetic testing to identify potential familial risk factors and to guide appropriate risk stratification and preventive strategies for all affected individuals.

## Discussion

This case highlights the diagnostic and management challenges associated with FAP, particularly when complicated by extracolonic manifestations such as incidental gastric tissue findings. Our 19-year-old male patient with a known family history of FAP presented with gastrointestinal symptoms that raised suspicion for colonic involvement. This was confirmed by colonoscopy, revealing multiple adenomatous polyps. These findings were consistent with typical colonic manifestations of FAP, a condition marked by APC gene mutations that lead to unchecked colonic epithelial proliferation and adenomatous polyp formation throughout the colonic mucosa. Given this pathophysiology, the adenomatous polyps observed in this patient originated from the colonic mucosa, reflecting the established etiology in FAP cases.

During histopathological analysis, an incidental gastric mucosal sample, likely representing a biopsy "floater," was observed, prompting *H. pylori* testing. Importantly, this sample did not influence the final diagnosis of colonic adenomas in the context of FAP, as no gastric-origin adenomas were identified. The presence of both chief and parietal cells within this extraneous gastric tissue initially raised the possibility of oxyntic adenomas, a rare type of gastric lesion characterized by dysplastic changes in the glandular epithelium. However, further examination ultimately supported the diagnosis of mixed hyperplastic/fundic gland polyps with focal low-grade dysplasia in the colonic adenomas, aligning with typical FAP pathology.

Oxyntic adenomas arise from the oxyntic mucosa (gastric corpus and fundus) and exhibit architectural disarray, nuclear atypia, and varying degrees of dysplasia, necessitating careful differentiation from other gastric neoplasms [1]. Conversely, mixed hyperplastic/fundic gland polyps with focal low-grade dysplasia, identified in our patient, are generally considered benign lesions featuring a combination of hyperplastic and fundic glandular elements with focal dysplastic changes. Histologically, these polyps present with elongated, tortuous, and dilated gastric pits, increased nuclear stratification, and occasional epithelial cell mitoses [1].

The distinction between these two pathologies is critical due to their differing clinical implications and management strategies. While oxyntic adenomas may require more aggressive surveillance and potential surgical intervention due to their malignant potential, mixed hyperplastic/fundic gland polyps are usually monitored for progression to dysplasia. In our case, however, the colonic location of the polyps and the incidental nature of the gastric tissue demonstrate the importance of accurate diagnostic assessment to avoid unnecessary concern regarding gastric adenomas and to direct appropriate monitoring and management strategies in FAP.

This case emphasizes the importance of a comprehensive diagnostic approach integrating clinical findings, endoscopic evaluation, and histopathological assessment. Accurate differentiation is vital in guiding the appropriate management of gastric polyps in FAP patients. Misdiagnosis could lead to either overtreatment or insufficient surveillance, both of which carry significant risks. FAP poses a significant risk of colorectal cancer due to the development of numerous adenomatous polyps in the colon and rectum. Managing FAP necessitates a comprehensive approach aimed at reducing cancer risk and improving quality of life. Regular endoscopies are crucial for monitoring polyp burden and detecting dysplastic changes early, with surveillance typically beginning in adolescence or early adulthood [7]. The timing and choice of surgical interventions, such as prophylactic colectomy, depend on factors like age, polyp burden, and patient preference. Options may include total proctocolectomy with ileal pouch-anal anastomosis, total colectomy with ileorectal anastomosis (IRA), or subtotal colectomy with IRA [8].

## Conclusions

This case of a 19-year-old male patient with recurrent lower abdominal pain, changes in bowel habits, and intermittent rectal bleeding highlights the intricate diagnostic challenges associated with FAP. Through a comprehensive evaluation involving clinical assessment, imaging studies, endoscopic examinations, and detailed histopathological analysis, we arrived at a diagnosis of mixed hyperplastic/fundic gland polyps with focal low-grade dysplasia. This case emphasizes the importance of distinguishing between various gastric polypoid lesions, particularly oxyntic adenomas and mixed hyperplastic/fundic gland polyps, due to their differing histological features and clinical implications. Accurate diagnosis is paramount in guiding appropriate management strategies and ensuring effective patient care. This case also illustrates that a comprehensive diagnostic approach is essential in navigating the complexities of FAP, ultimately contributing to improved patient outcomes and advancing our understanding of this hereditary condition.
